# Advanced Neck Dermatillomania Leading to Cervical Osteomyelitis and Epidural Abscess

**DOI:** 10.7759/cureus.48163

**Published:** 2023-11-02

**Authors:** Dia R Halalmeh, HusamEddin Z Salama, Petrica Molnar, Marc D Moisi

**Affiliations:** 1 Neurosurgery, Hurley Medical Center, Flint, USA; 2 Surgery, Michigan State University College of Human Medicine, Grand Rapids, USA

**Keywords:** skin picking, dermatillomania, cervical, epidural abscess, osteomyelitis

## Abstract

Dermatillomania, a condition characterized by compulsive skin picking, can lead to tissue damage and severe infections of adjacent structures. This case report presents the first documented instance of dermatillomania-induced cervical osteomyelitis and epidural abscess. Herein, we describe the case of a 45-year-old male patient with a history of a non-healing posterior neck wound, which progressively worsened and extended to the posterior cervical spine. The patient subsequently experienced weakness and paresthesia in the left arm. Neuroimaging revealed cervical spine osteomyelitis and an associated epidural collection/phlegmon compressing the spinal cord. The abscess was evacuated via posterior laminectomy, followed by culture-guided antibiotic therapy. The presence of a chronic wound or ulcer in the setting of psychiatric comorbidities should raise suspicion of dermatillomania-induced complications. Early diagnosis is essential to guide management and prevent serious complications. Management involves a multidisciplinary approach that includes addressing behavioral abnormalities and concurrent psychiatric disorders.

## Introduction

Dermatillomania, also recognized as psychogenic or neurotic excoriation, constitutes a distinct category within compulsive disorders. It is a relatively common behavioral condition, with a prevalence ranging from 1.4% to 5% in the general population [1,2]. According to the Diagnostic and Statistical Manual of Mental Disorders, Fifth Edition (DSM-5), patients exhibiting symptoms limited to body-focused repetitive behaviors (BFRB) are categorized under obsessive-compulsive and related disorders, which include conditions such as trichotillomania and nail biting [3]. While dermatillomania traditionally affects the face, it can frequently manifest in other areas of the body such as the neck, arms, shoulders, and fingernails [1,2]. Severe cases of dermatillomania often result in full-thickness wounds and, if left untreated, can lead to the development of skin infections over time. Of note, patients with dermatillomania may have other associated conditions, including anxiety disorder, obsessive-compulsive disorder, body dysmorphic disorders, and trichotillomania [1,2]; however, these conditions are not always present.

Osteomyelitis has been documented as a complication arising from pathological skin picking [4,5]. However, the reported infections have not previously been observed in the cervical spine. In this report, we present a unique case of a 45-year-old male exhibiting severe neck dermatillomania, resulting in the development of cervical epidural abscess and cervical osteomyelitis. To the best of our knowledge, this represents the first case of dermatillomania-induced cervical osteomyelitis and epidural abscess documented in scientific literature. The treatment approach involved posterior evacuation of the posterior epidural collection and debridement of the infected tissues. Despite providing comprehensive education on wound care and adherence to recommendations, the patient persisted in compulsive skin picking, leading to recurrent severe infections and exposure of the skull and cervical spine. This case highlights the critical role of multidisciplinary care, particularly the necessity of psychiatric evaluation, in managing patients with similar conditions.

## Case presentation

A 45-year-old male patient presented to the emergency department with a long-standing history of a non-healing posterior neck ulcer and recent paresthesia in the left arm. His medical history was notable for bipolar 1 disorder, anxiety disorder, and polysubstance abuse. He worked as a pig farmer. The wound had been present for nearly nine years, originating from a cyst on the posterior neck, which was surgically removed but later resulted in a methicillin-resistant *Staphylococcus aureus* (MRSA) infection. Despite treatment at a wound clinic, noncompliance with prescribed medications led to the development of a 1 × 2 cm ulcer on the left superior side of the nape of the neck, characterized by edema, granulation at the edges, and worsening pain. The patient underwent debridement and subsequent skin graft placement, with recommendations to continue wound care management at the clinic.

**Figure 1:**
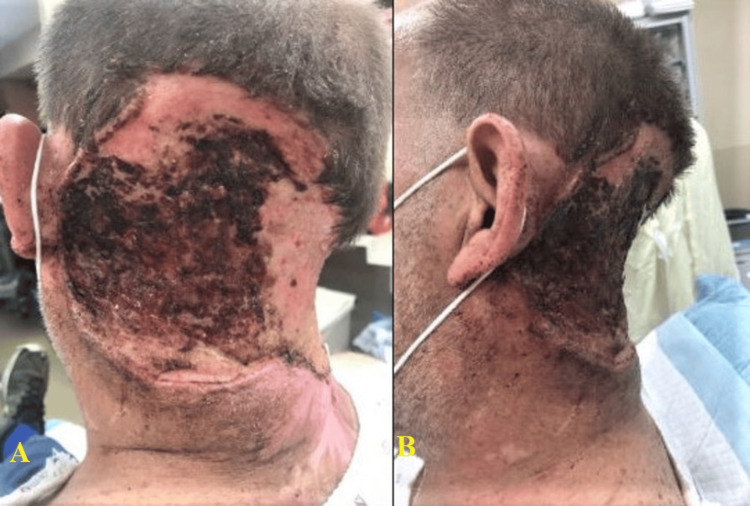
Large necrotic neck wound An extensive, deep, and relatively healing ulcer with black eschars can be seen. The lateral view demonstrates the depth of the lesion, progressing toward the cervical spine.

Due to his nonadherence to the prescribed treatment plan, the wound deteriorated due to recurrent infections, necessitating multiple debridements and skin grafts over several years. Recently, the patient reported paresthesia in the left arm and hand, affecting the fourth and fifth fingers, accompanied by weakness. Clinical examination revealed a large necrotic wound with foul-smelling, purulent discharge and noticeable blood oozing (Figure 1). Informed consent was obtained from the patient himself.

**Figure 2:**
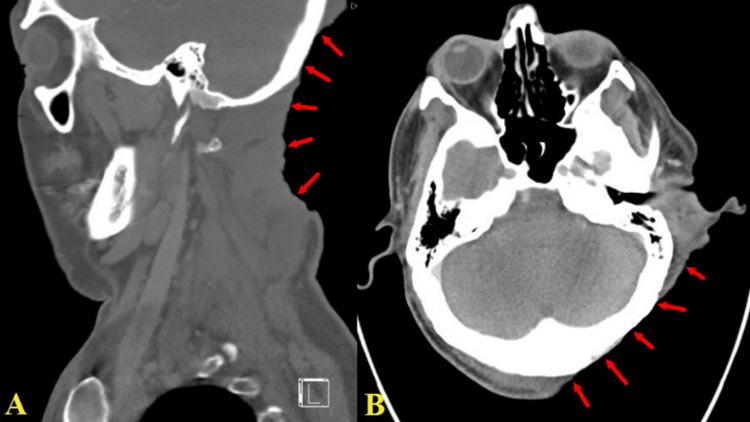
Large skin defect with occipital bone exposure Lateral head and neck (A) and brain (B) CT scan showing deep skin defect on the posterior neck reaching the occipital bones (red arrows). CT: computed tomography

Bacterial cultures confirmed the presence of MRSA in the tissue sample. Computed tomography (CT) scans and magnetic resonance imaging (MRI) of the patient's head and cervical spine were performed to evaluate the extent and severity of the wound and its deep-seated involvement. The CT scan revealed erosion of the mastoid processes, indicating chronic calvarial osteomyelitis and mastoiditis. Additionally, minimal cortical exposure was observed in the left occipital calvarium and mastoid region (Figure 2). MRI images displayed edema in the left suboccipital, retromastoid, and posterior paraspinal soft tissues, consistent with inflammation and infection. Phlegmonous changes and myositis were extensive, involving the left suboccipital paraspinal musculature down to the C5-C6 level; however, no evidence of osteomyelitis or discitis was noted at this level. The patient was subsequently hospitalized, receiving multidisciplinary care involving neurosurgery, plastic surgery, infectious diseases, and general surgery, alongside meticulous monitoring.

A comprehensive review of the patient's medical records revealed multiple emergency department admissions for blood transfusions due to severe anemia resulting from bleeding induced by razor blades. Additionally, drug-seeking behavior was frequently reported. During hospitalization, the patient was observed engaging in secretive removal of wound dressings and compulsive wound picking, expressing a need to eliminate perceived "bumps." Periodically, the wound was found to be intensely red and actively bleeding onto the patient's neck and shoulders, likely due to abrasion from utensils. Collaborating with the psychology team, the patient was educated about the vital importance of maintaining intact wound dressings before scheduled wound debridement and skin graft placement. Following surgical debridement, vacuum-assisted wound closure was employed, facilitating the patient's discharge home a few days later.

**Figure 3:**
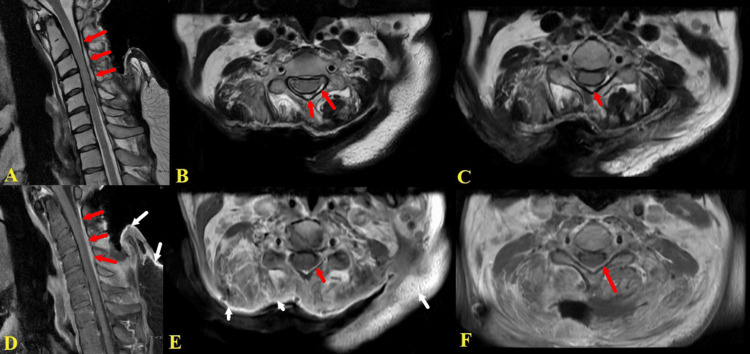
Posterior cervical osteomyelitis and epidural phlegmon T2 (A-C) and post-contrast T1 (D-F) weighted magnetic resonance images revealing posterior cervical epidural collection that enhances with contrast and extends from C2 to T1 (red arrows). Soft tissue signal abnormalities on contrast T1 are also noted, indicating inflammation due to infection (white arrows). Additionally, there is mild compression of the spinal cord caused by the epidural collection. Edges of the wound defect can also be appreciated.

At a subsequent examination, the patient presented with foul-smelling discharge and impaired wound healing. He complained of persistent neck pain and worsening weakness in the left upper extremity. Cervical MRI revealed heterogeneous enhancement in the soft tissue posterior to the neck spanning from C2 to C4. Signal abnormalities affecting the spinous processes of C2-C5 were indicative of spinal osteomyelitis. Furthermore, T2 and T1 contrast-enhanced images exhibited a collection in the posterior epidural space from C2 to T1, suggesting the presence of a posterior epidural abscess/phlegmon (Figure 3). To assess disease extension, an MRI of the thoracic and lumbar regions was obtained, but it showed no evidence of epidural abscess or inflammation in these regions.

**Figure 4:**
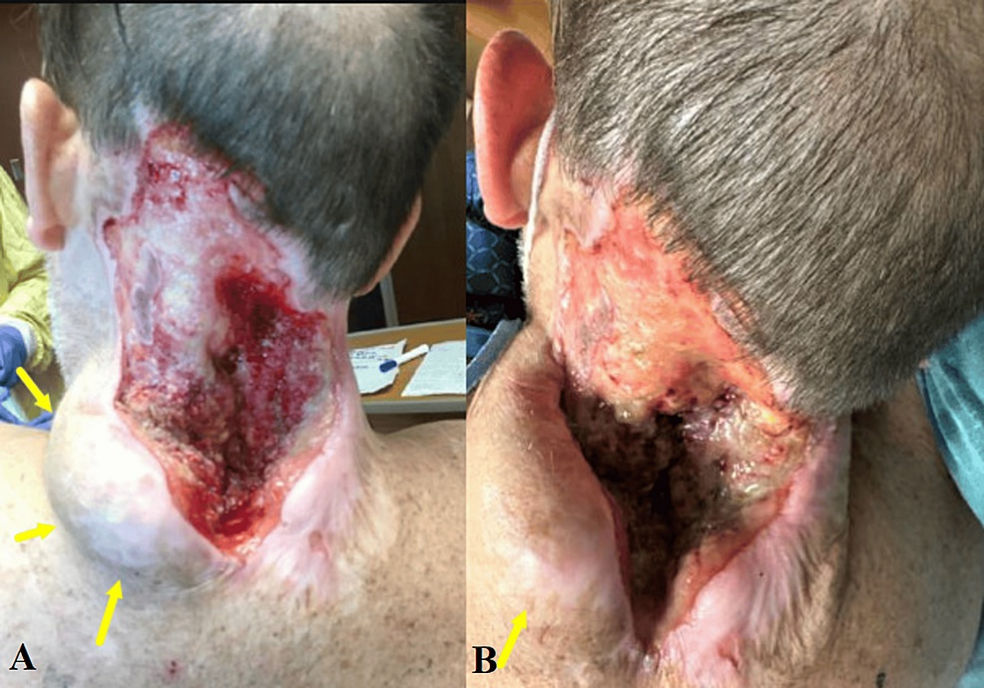
Wound infection associated with cervical osteomyelitis Photograph of the skin wound one month after surgical decompression and wound vacuum closure. A purulent necrotic base and localized swelling in the left lower edge (yellow arrows) can be seen.

Due to worsening neurological deficits, urgent surgical debridement of the epidural collection was planned. Prior to the surgery, the patient persisted in his skin picking behavior and occasionally consumed the removed skin fragments. A laminectomy at the C3-C4 level was performed. Careful dissection of the phlegmon from the dura mater was carried out, followed by thorough irrigation. Vacuum-assisted wound closure was employed. Tissue cultures yielded *Staphylococcus epidermidis*, which was appropriately treated with antibiotics. By postoperative day 2, the patient demonstrated symptom improvement. However, one month after the surgery, the patient presented to the emergency department with generalized weakness, fatigue, and chills. The wound appeared unchanged from previous examinations (Figure 4). The patient was advised to continue antibiotic therapy and attend follow-up appointments at the wound clinic for dressing changes. Additionally, efforts were made to optimize the management of his psychiatric comorbidities.

## Discussion

Dermatillomania, also recognized as skin picking disorder (SPD), is classified within the category of obsessive-compulsive and related disorders [6]. Like other anxiety and compulsive disorders, dermatillomania predominantly affects females, with a female-to-male ratio of approximately 8:1 [7]. It commonly emerges during adolescence or early adulthood, with a mean age of onset ranging between 30 and 40 years [6]. The prevalence of this disorder in the general population varies from 1.4% to 5.4% [2,7,8]. Dermatillomania is frequently accompanied by other psychiatric comorbidities; however, it can also manifest in isolation. In the presented case, the patient had a psychiatric history notable for bipolar 1 disorder and anxiety disorder. In addition, our patient's medical history of polysubstance abuse may have contributed to his current condition by exacerbating the psychiatric comorbidities.

The diagnosis of dermatillomania involves a comprehensive assessment conducted by a mental health professional, typically a psychiatrist or psychologist. The diagnosis relies on specific criteria outlined in the Diagnostic and Statistical Manual of Mental Disorders, Fifth Edition (DSM-5), encompassing repetitive skin picking behavior resulting in skin lesions, multiple unsuccessful attempts to cease or reduce the behavior, and notable distress or impairment in daily functioning as a consequence of the behavior [6]. It is crucial to exclude other medical or psychiatric conditions that might contribute to the behavior, such as dermatological disorders. In some patients, it may be hard to differentiate dermatillomania from other skin or autoimmune disorders. Therefore, obtaining a thorough history is an important step to exclude other conditions. Nevertheless, the presence of repetitive skin picking behavior (rather than hair pulling, which is seen in trichotillomania) in the context of relevant medical history makes dermatillomania more likely. Moreover, trichotillomania is typically associated with patchy hair loss and regrowth of new hair shafts of various lengths (not seen in our patient).

Dermatillomania can lead to various complications, including scarring, infections, chronic pain, bleeding, and disfigurement [4]. Psychologically, the disorder can cause significant distress, anxiety, social isolation, and feelings of shame or embarrassment [1]. Often, seeking medical help is hindered by social embarrassment or a lack of awareness regarding the effectiveness of treatment. Psychologists and psychiatrists are the primary healthcare providers for these patients [1], but other specialties such as dermatology and neurosurgery may be consulted for skin complications and severe infections. In the present case, the patient exhibited severe neck dermatillomania resulting in recurrent skin infections, ultimately leading to posterior cervical osteomyelitis and epidural abscess, necessitating surgical intervention due to neurological deficits.

Cervical osteomyelitis is an uncommon infection of the bone and adjacent soft tissues of the cervical spine. Spinal epidural abscess is a frequent sequela of vertebral osteomyelitis [9]. The infection can extend to the adjacent neural elements (e.g., epidural space) leading to cervical epidural abscess, a severe complication with an estimated incidence of 0.2-2.8 per 10,000 hospital admissions [10]. The primary predisposing factors often include conditions that compromise the immune system, such as diabetes mellitus, immunosuppression, and intravenous drug abuse [11]. Despite the patient's history of polysubstance abuse, this case represents, to the best of our knowledge, the first reported instance of dermatillomania-induced cervical osteomyelitis and epidural abscess.

Repetitive skin picking and scratching create breaches in the skin barrier, allowing bacteria to infiltrate and infect the underlying tissues. Spinal epidural abscess (SEA) is a rare yet potentially severe infection of the epidural space, necessitating prompt diagnosis and treatment to prevent permanent neurological damage or mortality. SEA should be considered in any patient displaying the following signs and symptoms: localized tenderness over the affected spinal area, focal neurological deficits (such as radiculopathy or weakness), and systemic manifestations (such as fever, chills, and elevated inflammatory markers, specifically C-reactive protein (CRP)). Suspicion is heightened in individuals with additional risk factors, such as intravenous drug use, advanced age [12], and patient-specific factors, as illustrated in the current case where compulsive skin picking played a significant role. Such presentations warrant immediate neuroimaging evaluation to exclude serious spinal cord compression. MRI with contrast is the preferred imaging modality because it is often positive in the early stages of the disease, offering optimal visualization of the infection's location and extent [13].

The primary objective of treatment is to completely evacuate any collections and eliminate the underlying bacterial infection through surgical intervention and intravenous antibiotics, respectively. Surgical intervention typically involves decompressive laminectomy, debridement, and pus drainage, performed once the diagnosis is confirmed in clinically stable patients without paralysis occurring within the initial three days of onset [10]. Even if paralysis is evident, surgical decompression and evacuation may be considered to prevent further infection extension and subsequent systemic dissemination. In our case, the patient exhibited focal neurological deficits attributed to cervical epidural abscess with spinal cord compression, necessitating neurosurgical intervention. Notably, failure to respond to initial antibiotic therapy in patients lacking neurological deficits may indicate an early sign of epidural collection. Although mortality rates related to SEA have decreased, one study reported 5%-10% of patients succumbing to sepsis, meningitis, and related complications [10,14]. Consequently, infectious disease specialists should always be consulted for guidance and ongoing microbial management.

In our case, a posterior approach was employed for surgical intervention, and cultures from the abscess identified *Staphylococcus epidermidis*. The patient responded positively to treatment and was discharged with appropriate antibiotics, with scheduled outpatient follow-up. Given the rarity of dermatillomania-induced cervical osteomyelitis and epidural abscess, this possibility should be considered in patients with a significant history of polysubstance abuse, anxiety, and mood disorders characterized by tendencies toward compulsive-impulsive behaviors. Furthermore, comprehensive psychiatric evaluation is crucial for the effective management of patients exhibiting severe manifestations of skin picking disorder. Overall, a multidisciplinary approach and comprehensive counseling emphasizing the importance of behavior cessation are integral components of the management plan.

The multidisciplinary approach involves the collaborative efforts of psychiatrists, dermatologists, ID specialists, and potentially surgeons (for surgical complications) or the oversight of a specialist with experience in both psychiatric and dermatological disorders. Cognitive-behavioral therapies, including habit reversal therapy, acceptance-enhanced behavior therapy, and cognitive-behavioral therapy, have demonstrated improvements in skin picking disorder (SPD) based on observational studies and randomized trials [1,15]. Clinician-guided self-help therapy through internet-based programs has also exhibited promising outcomes [16]. Pharmacological interventions, such as antidepressants, antipsychotics, glutamate-modulating drugs, and anxiolytics, have been explored, but their effectiveness for SPD remains uncertain. Selective serotonin reuptake inhibitors, a class of antidepressants, have shown some benefits, although their evaluation in SPD is limited to a few clinical trials [17]. Atypical antipsychotics may be beneficial for patients with delusional symptoms [18], and glutamate-modulating drugs such as N-acetylcysteine have demonstrated partial efficacy [19]. In cases where anxiety disorder underlies SPD, anxiolytics could be considered as a treatment option, even in the absence of specific studies demonstrating their effectiveness for SPD. Unfortunately, many patients lack insight into their condition, leading to the progressive worsening of compulsive behavior. This complicates overall management and heightens the risk of severe complications, as observed in our patient who was resistant to psychological counseling. Close monitoring with frequent follow-up visits is essential to ensure adherence to recommendations, proper wound care management, and prevention of unnecessary complications. Similar to refractory cases of obsessive-compulsive disorders, more invasive interventions such as deep brain stimulation could be considered. However, the effectiveness of such interventions for dermatillomania remains uncertain due to the rarity of this condition.

Effective wound healing is pivotal to halting further disease progression. When SPD leads to secondary skin infections, physicians may prescribe topical and/or systemic antibiotics to address excoriations or ulcerations. Semi-occlusive dressings might be recommended to minimize additional skin damage and facilitate the healing process. In this study, the patient underwent vacuum-assisted wound closure to enhance wound healing. To alleviate pruritus, potent topical corticosteroids or intralesional glucocorticoids may be employed, although their efficacy is primarily based on clinical experience. Phototherapy utilizing narrowband ultraviolet B has demonstrated benefits in reducing pruritus and skin-picking behaviors in specific patients [20]. Finally, further research with a larger patient population, alongside prospective data collection and long-term follow-up, is essential to identify the specific characteristics of individuals with dermatillomania who are prone to developing wound complications or severe infections. Pharmacological interventions have been utilized in the treatment of conditions sharing obsessive-compulsive characteristics, and they can be considered for patients with dermatillomania. Nevertheless, the clinical effectiveness of pharmacological treatments may be limited without a comprehensive multidisciplinary approach, including focused psychiatric evaluation and behavioral therapies. Complete symptom resolution may be challenging to achieve solely through medication. Consequently, future research efforts should explore these medications in conjunction with ongoing behavioral therapy, preferably through comparative analyses, to evaluate their combined efficacy.

## Conclusions

Dermatillomania-induced skin and soft tissue infections can lead to significant morbidity and even death. The resultant vertical progression of infection can lead to serious complications including cervical osteomyelitis and epidural abscess. This article represents the first documentation of spinal epidural involvement attributed to dermatillomania. Patients with comorbid psychiatric conditions and polysubstance abuse seem to be vulnerable to severe complications of dermatillomania. The presence of neurological deficits should prompt urgent imaging and evaluation for spinal cord involvement, which requires surgical evacuation and debridement. Management entails a multifaceted approach, encompassing potent antibiotic therapy, meticulous wound care, and collaborative engagement with diverse specialties, particularly neurosurgery and infectious diseases. Concurrently, addressing underlying psychiatric comorbidities is indispensable for comprehensive patient care.
